# Role of the Pinning Points in epitaxial Graphene Moiré Superstructures on the Pt(111) Surface

**DOI:** 10.1038/srep20354

**Published:** 2016-02-08

**Authors:** José I. Martínez, Pablo Merino, Anna L. Pinardi, Otero-Irurueta Gonzalo, María F. López, Javier Méndez, José A. Martín-Gago

**Affiliations:** 1 ESISNA Group, Dept. Surfaces, Coatings and Molecular Astrophysics, Institute of Material Science of Madrid (ICMM-CSIC), Sor Juana Inés de la Cruz 3, 28049 Madrid, Spain; 2 Center for Astrobiology (INTA-CSIC), Torrejón de Ardoz, 28850 Madrid, Spain; 3 Center for Mechanical Technology and Automation (TEMA), University of Aveiro, 3810-193 Aveiro, Portugal

## Abstract

The intrinsic atomic mechanisms responsible for electronic doping of epitaxial graphene Moirés on transition metal surfaces is still an open issue. To better understand this process we have carried out a first-principles full characterization of the most representative Moiré superstructures observed on the Gr/Pt(111) system and confronted the results with atomically resolved scanning tunneling microscopy experiments. We find that for all reported Moirés the system relaxes inducing a non-negligible atomic corrugation both, at the graphene and at the outermost platinum layer. Interestingly, a mirror “anti-Moiré” reconstruction appears at the substrate, giving rise to the appearance of *pinning-points*. We show that these points are responsible for the development of the superstructure, while charge from the Pt substrate is injected into the graphene, inducing a local *n*-doping, mostly localized at these specific *pinning-point* positions.

Graphene growth on metal surfaces is one of the most promising routes towards scalable production of high-quality graphene^1^. For this purpose the growth of graphene is carried out on surfaces where the substrate plays a double role: first, as a catalyst; and second, as an *easy-to-remove* platform. Several substrates are routinely used in the search for high-quality *free-standing* graphene single layers, among which transition metal (TM) surfaces represent a good model system where to rationalize their basic properties^2^,^3^. Graphene grows on single crystal TM surfaces normally forming large, extended and faultless domains, permitting to test the effects of i.e. electronic doping, molecular adsorption, intercalation reactivity or growth dynamics^2^,^3^,^4^.

The interaction between high-symmetry metal substrates and graphene varies from weak physisorption to strong chemisorption^5^ depending on the supporting TM surface. On one hand, on highly interacting substrates – such as Ru(0001)^6^,^7^ or Ni(111)^8^ – the atomic structure is generally well described. As an example, in the Gr/Ru(0001) system it is well known that the graphene structure consists of a highly corrugated network of nanodomes surrounded by regions of high Gr—Ru interaction. On the other hand, the exact determination of the atomic structure of epitaxial graphene on weakly interacting TM substrates – such as Ir(111)^9^,^10^,^11^,^12^,^13^, Cu(111)^14^, Pd(111)^15^ or Pt(111)^16^,^17^,^18^,^19^,^20^ – turns into a very challenging task, since normally a large number of rotational domains forming different Moirés coexist on the same single crystal surface and can form polycrystalline graphene domains. Therefore, a microscopic characterization of their different adsorption geometries is needed.

Although there is a large amount of works devoted to the study of the Moiré structures^6^,^7^,^8^,^9^,^10^,^11^,^12^,^13^,^14^,^15^,^16^,^17^,^18^,^19^,^20^,^21^,^22^, there are not many systematic studies determining all the emerging experimental superstructures appearing in Gr/TM systems. Recently, our group has proposed a simple model to study Gr/TM(111) systems^19^. This phenomenological model describes the stability of a Moirés on TM(111) surfaces taking only into account the lattice parameters of substrate and graphene, and yielding, as an example, a large number of different Moiré patterns (22) for the Gr/Pt(111) system. Comparing these predictions with the structures observed in our scanning tunneling microscopy (STM) sessions (as well as the previous available literature) we find a rather surprising agreement. Besides, an elegant geometrical model has been proposed by K. Hermann to predict Moiré patterns of graphene on hexagonally-packed metal surfaces through their spatial beating frequencies^23^. This solution successfully predicts, as the one proposed by Merino *et al.*^19^, some of the Moiré domains analyzed in the present study.

It is important to remark that the aforementioned phenomenological model only takes into account very simple geometrical considerations. The outcome rises from the numerical search of the best matching pair of C—Pt atoms for any given rotational angle, considering any lateral relaxation within the system negligible. Surprisingly, the determination of the minimum mismatch exclusively for a pair of atoms describes the stable domain orientations in the Gr/Pt(111) system. The main consequences of this experimental observation are noteworthy. The fact that minimization of the mismatch exclusively at the coincidence point (rather than minimization of the strain over the whole unit-cell) yields the best predictions introduces a novel scenario where most of the interaction must be ruled by the coincidence of some particular atoms within the Moiré unit-cell. These regions would act as sort of *pinning-points* for the Moiré superlattice, whilst the rest of the graphene layer interacts weakly with the metallic substrate, and can be considered unaffected by the metal underneath^22^. Moreover, the question of the dependence of charge carrier doping on the rotation angle of the Moiré is still open.

To better understand all these effects, a characterization by means of first-principles density functional theory (DFT) of the structural and electronic properties of the different Moirés becomes necessary. However this turns into a very challenging task due to large size of the unit cells involved. To this aim, in the present work we analyze the nature of the graphene—substrate interaction for several Moiré superstructures appearing for Gr/Pt(111) by using an adequate combination of local ultra-high vacuum (UHV) STM experiments and first-principles calculations, accounting for an accurate – given the high amount of atoms involved in some of the calculations – van der Waals (vdW) interaction. We show hereafter that the Pt surface atoms tend to relax out of plane, approaching towards the graphene layer and originating a sort of low-dimensional draining points where charge can efficiently flow between the two materials.

## Methods

### Experimental section

Experiments were carried out in an ultra-high vacuum (UHV) chamber with base pressure of 1 × 10^−10^ mbar equipped with low energy electron diffraction (LEED) optics and STM at room temperature (RT). The temperature of the samples was monitored by an IR pyrometer with an emissivity of 0.25. The samples were cleaned by the conventional procedure of repeated cycles of argon sputtering and annealing (at 1200 K for 20 min). The first annealing cycle was performed in an oxygen atmosphere (P = 1 × 10^−6^ mbar) to remove major carbide impurities. The sample surface cleanliness was checked by STM and LEED prior to the graphene growth. Commercial C_60_ (Sigma, 98% purity) was deposited at a rate of 0.4 monolayers per hour during 30 min, keeping the sample at room temperature. The samples were then annealed by electron bombardment at temperatures up to 1200 K for 5–10 min intervals. Importantly, during this procedure the pressure did not exceed 5 × 10^−10^ mbar. Submonolayer coverage of flat and homogeneous graphene islands was observed on top of clean Pt regions and, thus, modifying the deposition time the total coverage can be controlled. A long deposition time leads to complete monolayer coverage. STM images were recorded using topographic and current modes with typical biases of −250 to 250 mV and currents ranging between 0.1 and 4 nA. WSxM software was used for data acquisition and image analysis^24^. Thermal drift was corrected using a custom program in order to reduce errors in measurements of angles and distances. This program corrects the images for a given unit cell keeping the fast scan axis distances as the reference one.

### Computational details and theoretical approach

We have used two different atomistic simulation packages: i) the plane-wave code PWSCF^25^ used for the characterization and optimization of the atomic configurations, by accounting van der Waals forces; and ii) the localized basis set code FIREBALL^26^ used for the theoretical STM imaging calculations. Technical details concerning both mentioned approaches have been explained in detail elsewhere^25^,^26^, and here we will only summarize the main points of both first-principles implementations.

Thus, as starting point we use the atomic configurations predicted by the phenomenological model. Commensurate unit cells have been used to perform the calculations; while the geometrical model neglects relaxations and does not account for the commensurability of the system. However, the mismatches between commensurate and the incommensurate superstructures (predicted models) are small^19^ and the use of commensurate structures is an imperative in DFT calculations. The atomic geometries have been fully relaxed by means of DFT calculations by including dispersion forces within the DFT+D approach^27^ as implemented in the PWSCF^25^ in a supercell approximation. For this purpose, we have used the revised version of the generalized gradient corrected approximation of Perdew, Burke, and Ernzerhof (rPBE)^28^, and an empirical efficient vdW R^−6^ correction to add dispersive forces to conventional density functionals – see details in previous literature^27^,^29^ –. To justify the use of this vdW implementation in the calculations, it is important to remark that there is strong evidence that van der Waals (vdW) – or dispersion forces – play an paramount role in the adsorption mechanism of aromatic molecules on metal surfaces^30^,^31^,^32^. In many aspects, graphene can be understood as an extended aromatic system, given the existence of π electronic clouds above and below the graphene basal plane. In this line, the important role of dispersion forces in graphene on metal surfaces has been already reported in literature^6^,^7^,^31^,^33^, leading, in comparison with non-vdW DFT calculations, to a significant increase of the adsorption energies and distances, and, what is even more relevant, to non-negligible geometric distortions of both the graphene and the metal substrate^6^,^34^. Recently, Carrasco and coworkers have exhaustively investigated the performance of a large variety of vdW-inclusive DFT schemes to compute adsorption properties of Bz on (111) surfaces of Cu, Ag, Au, Rh, Pd, Ir, and Pt, concluding that optimized exchange functionals (optPBE, optB88, and optB86b) provide the most accurate results^35^. The large amount of atoms involved in some of our systems prevents us of using such accurate approaches. Nevertheless, DFT+D vdW-inclusive frameworks have demonstrated to provide a fairly good performance yielding excellent results as compared to the experimental evidence^6^,^7^,^31^,^33^,^34^. To check the validity of our results we have additionally performed, for the 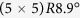 and the 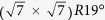 Gr/Pt systems, localized-basis set + vdW calculations within the dipolar approximation^26^,^36^ and DFT+D calculations by using the local density Perdew-Zunger parametrization^37^ as underlying conventional XC functional, obtaining with both vdW schemes a good comparison with those presented in this study, which justifies our choice of this DFT+D vdW framework.

The ion–electron interaction is modeled by ultrasoft pseudopotentials^38^, and the one-electron wave-functions are expanded in a basis of plane-waves, with energy cut-offs of 400 and 500 eV for the kinetic energy and for the electronic density, respectively.

### Theoretical STM imaging

STM simulations have been performed for all the Gr/Pt(111) Moiré configurations considered in this study, to be compared with the experimental UHV-STM images. In order to obtain STM images and tunneling currents, we used an STM theoretical simulation technique that includes – by construction – a detailed description of the electronic properties of both the tip and the sample. Using this technique, based on an effective combination of a Keldysh—Green formalism and local orbital density functional theory^39^ we split the system into sample and tip, where the samples here are the different Gr/Pt(111) systems. In these calculations we have assumed to simulate the scanning with a W-tip formed by 5 protruding atoms (one of them in the apex) attached to an extended W(100)-crystal. We have reported on the effect of the tip geometry in the theoretical STM images within the Keldysh—Green formalism^40^. Using such a sharp model tip with just one terminating atom in the apex has demonstrated to maximize the tip-sample orbital overlapping during the scanning in our calculations and provides excellent comparison to the experimental evidence^31^,^32^,^40^.

## Results and Discussion

We have performed a quantitative theoretical characterization of five of the most stable and representative Moiré superstructures predicted by our aforementioned geometrical model^19^, by using, as explained, two different atomistic simulation packages: the plane-wave code PWSCF^25^ used for the structural optimization of the atomic configurations accounting van der Waals forces; and the localized basis set code FIREBALL^26^ for the theoretical Keldish-Green STM imaging calculations. These structures were modeled using single graphene sheets over 4 layers of Pt(111), enough to guarantee convergent results and reproduce the clean Pt surface. Given the large amount of atoms involved in all the calculations we have limited the depth of the substrate slab to mimic the Pt(111) surfaces to four metal layers, keeping fix in the relaxations the two bottommost layers. Nevertheless, additional calculations by including an extra Pt layer reveal no significant variations in the adsorption energies (with an estimated uncertainty of 0.01 eV) or in the electronic structure of the topmost layer. Additionally, the corrugation exhibited by the topmost Pt layer does not extend to any of the deeper metal layers, which can be considered as negligible. For all the cases, we have considered a minimum distance of ~25 Å of vacuum between neighbouring cells along the axis perpendicular to the surface, as well as full periodic boundary conditions representing infinite Gr/Pt interfaces. The periodic supercells of the selected superstructures were adjusted to 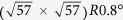, 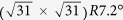, 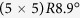, 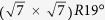, and 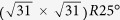, which correspond to μ, ζ, ε, β and ζ Gr/Pt Moiré superstructures, respectively (according to the nomenclature used in refs 19,41. The Brillouin zone (BZ) was sampled by means of [4 × 4 × 1], [2 × 2 × 1] and [1 × 1 × 1] Monkhorst-Pack grids for the 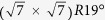, 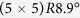 and 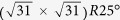 and 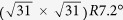, and 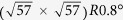 configurations, respectively.

**Figure 1:**
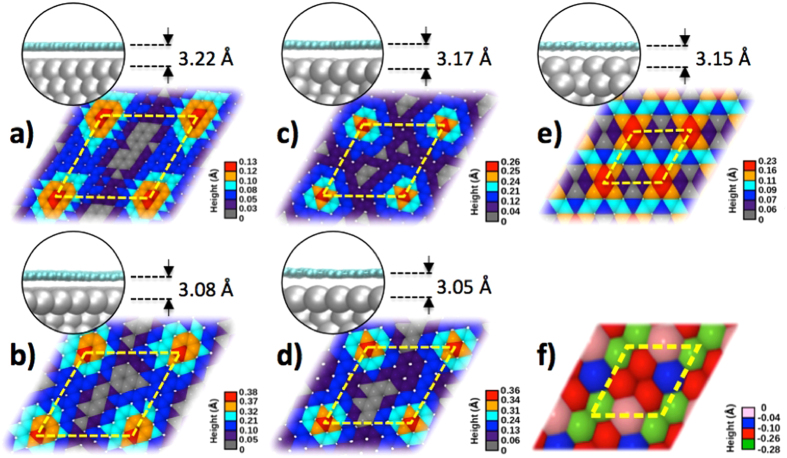
Optimized ground-state geometries of the five Gr/Pt(111) Moiré superstructures considered in this study: (a) 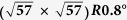 or μ_R0.8°_, (b) 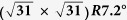 or ζ_R7.2°_, (c) 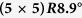 or ε_R8.9°_, (d) 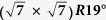 or β_R19°_, (e) 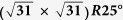 or ζ_R25°_. C atoms of the graphene monolayer are depicted in different colors depending on their relative height (the lowest C atom is set as a baseline at 0 Å). (**f**) Topmost Pt layer “anti-moiré” pattern for the β_R19°_ case is also shown (the topmost Pt atom is set as a baseline at 0 Å). Light-colored dashed lines indicate the unit cell used in the calculations. Side views of the Gr/Pt(111) structures are also included showing the minimum perpendicular distance between the Pt(111) surface and graphene.

Figure 1a–e show the optimized ground-state geometries for the different Gr/Pt(111) rotational domains considered. The height color-map used to depict the C atoms in each graphene monolayer represents the out of plane corrugation appearing in all the structures. The first important feature that we observe is that the relaxation of the different graphene Moirés has a reflection in a non-negligible corrugation of the Pt(111) topmost layer, inducing the formation of a complementary “anti-Moiré” out of plane displacement of the surface Pt atoms -with structural corrugations of up to 0.34 Å for the 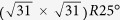 structure. Thus, the lowest carbon atom in the graphene layer coincides with the highest atom in the Pt surface; leading to the formation of a *pinning-point* just at the perpendicular coincidence between a C atom “on top” a Pt atom. The calculated Pt surface relaxation, induced by the interaction with the graphene, manifests the high malleability of Pt under these *a-priori* soft interactions. The region affected by the structural relaxation involves 2 Pt and 4 C atoms for the structure 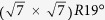, as it is shown in Fig. 1f.

Table 1 summarizes the values of the most important quantities extracted from the structural characterization for all the Moiré superstructures shown in Fig. 1. As reported in previous literature for other similar systems^6^,^7^, it is important emphasizing the crucial role played by the vdW forces in all the quantities shown in Table 1. The aforementioned vdW-DFT framework (DFT-D)^26^ is known to lead to a significant increase of the adsorption energies and distances of around 40% and 10%, respectively^6^,^7^, which also occurs in the present study. Besides, from the data reported in Table 1 it is possible to extract some interesting correlations. The average weighted perpendicular distance between the Pt surface and the graphene sheet, *d*, takes its highest value of 3.23 Å for the configuration 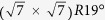, the domain with the highest occurrence frequency in the experiment. All the weighted adsorption distances shown in Table 1 range between 3.13 and 3.23 Å (higher than 3 Å), which contrasts with that observed for the Gr/Ru surface of 2.2 Å^6^, revealing a different adsorption character between both systems, associated with a higher reactivity of the Ru(0001) surface compared with Pt(111). Interestingly, these adsorption distances scale with the charge transfers occurring from the Pt towards the graphene for all the configurations (see below). On the other hand, the strongest adsorption energy (per C atom) of 0.29 eV is found for the 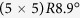 structure. Nevertheless, a non-significant variation between the adsorption energies per C atom is observed for the different structures, which range between 0.24 and 0.29 eV/C, to be compared to that obtained for the strongly interacting Gr/Ru interface of around 0.4 eV/C^6^, or those for the weakly Gr/Ir(111)^22^ systems of around 0.05 eV/C, respectively. Moreover, a partial scaling can be found between the charge transfer and the graphene Dirac-cone shift w.r.t. non-deformed graphene on Pt(111), with the exception of the domains where the charge transfer is more pronounced, the 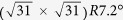 and 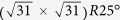 structures. This effect will be analysed in detail below.

By inspecting the structural ripple heights (see Table 1) observed in the graphene sheets for all the Moiré superstructures (ranging between 0.13 and 0.38 Å), no correlation with any of the other quantities can be easily extracted. Nevertheless, the non-negligible structural corrugations found in the relaxed Pt surfaces (ranging between 0.02 and 0.34 Å) take higher values with an increasing rotation angle. The low values of the graphene corrugations contrast with those observed for the Gr/Ru surface ranging between 1.2 and 1.6 Å^6^,^42^,^43^, where a substantially stronger interaction induces a more pronounced deformation of the graphene sheet. It is important to notice that if we consider the total structural corrugation – defined as the sum of the graphene and Pt corrugations –, which is maximum at the atomic positions of the mentioned *pinning-points*, this quantity does increase for increasing rotation angles. At 30°, the maximum possible angle, our group has reported the existence of an atomic vacancy network in the 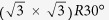 Moiré pattern of the G/Pt(111)^44^. This extremely corrugated topmost Pt slab can be seen as a limit case of the corrugation-rotation correlation.

**Figure 2:**
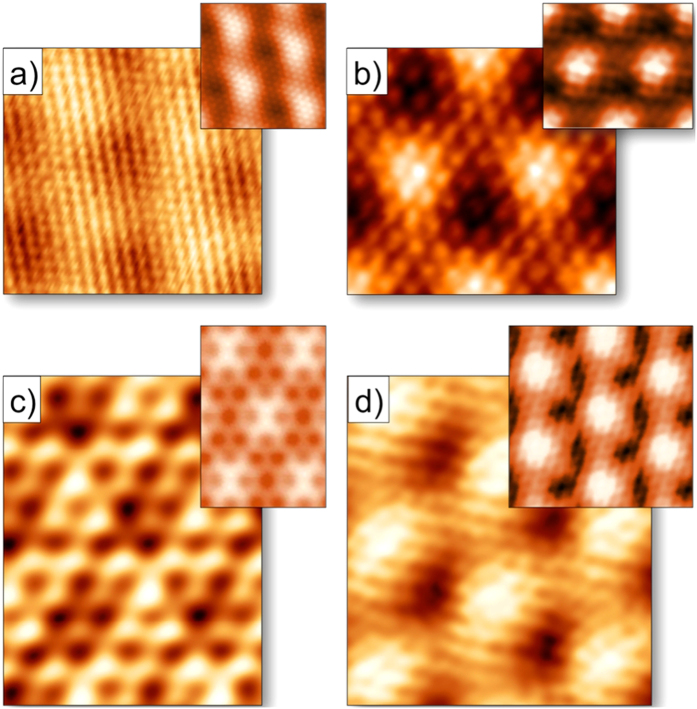
Comparison between UHV experimental (large pictures) and theoretical (top-right corner insets) constant-current STM images for the (a) μ_R0.8°_, (b) ε_R8.9°_, (c) β_R19°_ and (d) ζ_R7.2°_ Gr/Pt(111) Moiré superstructures (see Fig. 1) for *V*_*s*_ = −0.2–0.2 V and *I*_*tunnel*_ = 0.1 nA.

The simulated constant-current STM topographic images are in rather good agreement with the experimental ones in the range of V_s_ from −0.5 to 0.5 eV. As an illustration, Fig. 2 shows a comparison of STM images for V_s_ up to 0.2 V. It can be seen that the periodic Moiré pattern in the STM image is well reproduced by theory, and not only the relative intensity but also the apparent size of the different STM features as valleys (corresponding to topographically lower areas) and brighter zones (corresponding to topmost regions of the graphene Moirés).

**Figure 3:**
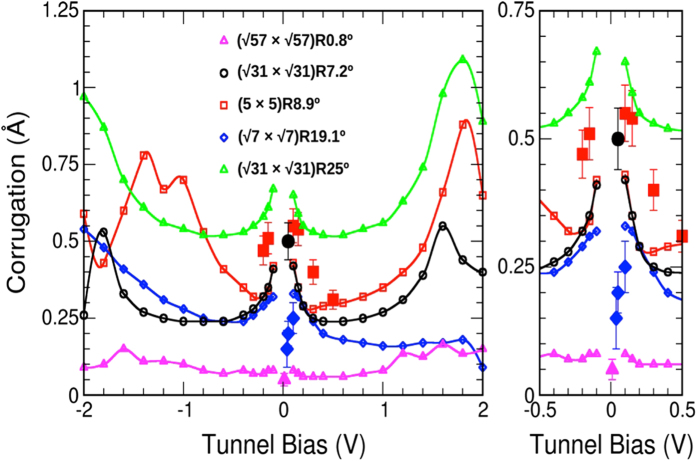
Experimental (solid symbols) and computed (empty symbols) apparent Moiré corrugation (in Å) as a function of the tunnelling bias voltage (in V) for the different Gr/Pt(111) superstructures shown in Fig. 1 along the same selected scanning paths. Theoretical STM images were obtained at constant-current conditions with *I*_*tunnel*_ = 0.1 nA mimicking the experimental tunnelling conditions.

A more stringent test is to compare STM profiles of apparent height (corrugation) along specific directions as the tunnel voltage varies. Figure 3 shows the variation of the apparent corrugation, 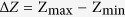, with *V*_*s*_ in the interval [−2.0, +2.0] V along the same selected scanning paths. The experimental results were obtained at room temperature in different experimental STM sessions, involving different tips (W-etched) and measuring conditions. The criteria for tuning the bias, current and feedback parameters are to maximize the resolution within the graphene superstructures. The overall variation with the tunnel bias is fairly well reproduced by theory for all the Gr/Pt structures. In particular, for the case of the 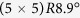 Moiré superstructure (with more experimental data-points available) both experiment and theory predict that the apparent corrugation strongly vary in the proximity of the Fermi level, from 0.3 to 0.6 Å in the voltage window [−0.5, +0.5] V. Experimental data-points shown in Fig. 3 were acquired by tracing profiles on the images and searching the mean maximum height difference. On the other hand, from the calculated profiles we observe that all the structures exhibit an almost constant corrugation up to voltages of around −1 and +1 V, except the 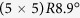 structure, which exhibits a noticeable changing behavior between −2 and 0 V. Below and above −1 and +1 V, respectively, a region with increasing corrugation is observed for all the structures. As a general trend, the Gr/Pt Moirés with a lower rotation angle with respect the Pt substrate show lower values of corrugation.

**Figure 4:**
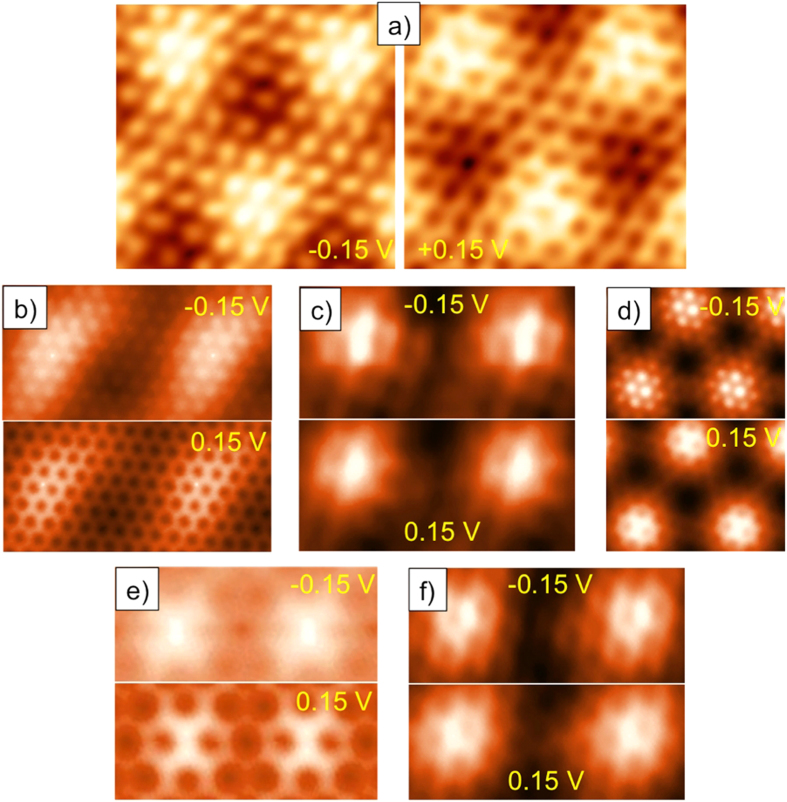
(**a**) Experimental STM images corresponding to the 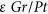 Moiré for *V*_*s*_ = −0.15 and 0.15 V, with *I*_*tunnel*_ = 0.1 nA. Theoretical constant-current STM images of Gr/Pt(111) for *V*_*s*_ = −0.15 and 0.15 V with *I*_*tunnel*_ = 0.1 nA, for the Moiré superstructures shown in Fig. 1: (**b**) μ_R0.8°_, (**c**) ε_R8.9°_, (**d**) ζ_R25°_, (**e**) β_R19°_ and (**f**) ζ_R7.2°_.

An interesting STM bias-dependent morphology behavior has been recently evidenced in the experimental STM images. Some of the experimentally observed Gr/Pt Moiré superstructures present a visible change in the STM contrast for different tunnel voltages (see an example for the ε Gr/Pt Moiré at *V*_*s*_ = −0.15 and 0.15 V in Fig. 4a), which is also observed in the calculated STM images of some of the superstructures. Figure 4b–f show the calculated STM images at constant-current regime (*I*_*tunnel*_ = 0.1 nA) for all the Gr/Pt structures considered in this study (see Fig. 1) for tunnel voltages of −0.15 and 0.15 V. In the STM images simulated over occupied states – at a negative voltage of −0.3 V – we can observe the atomic graphene lattice consisting of balls located on the aromatic rings forming a sort of “hill-pattern”, which emerges within the overlaid super-periodicity of the Moiré.

Nevertheless, when tunneling is carried out over unoccupied states – at a positive voltage of +0.3 V – the “hill-pattern” visible for negative voltages turns into a “hollow-pattern”, where each dark hole is located again on hollow sites of the graphene sheet, just where the bright balls where located at negative voltages. This interesting behavior may be explained in terms of the local doping level of the graphene layer for each, where the Dirac-cone is shifted w.r.t. pristine graphene, breaking its characteristic electronic symmetry around the Fermi energy. This doping-induced shift makes that tunneling at negative or positive voltages, close to Fermi level, yields different STM morphological contrasts. A similar STM contrast switch effect has been observed for the Gr/Ir surface by effect of different scanning tips used^10^.

With all this structural information, we are now in a position that permits us to discuss the electronic behavior of the different Moirés. Although the effect of the doping level in the Gr/Pt(111) interface has been previously reported^45^, it has been globally averaged for the whole multi-domain Gr/Pt structure, without considering the effect in each particular rotational domain (as well as for the case of the Gr/Cu/Ir(111) interface^46^). To shed some light on the level of local doping in the individual Gr/Pt Moirés, we have computed, for the Moirés analyzed in this study (see Fig. 1), charge transfers and density of state (DOS) profiles projected on the different graphene sheets. This electronic information allow us to analyze the character of each interfacial interaction, as well as the relative shift of the Dirac-cone for the different graphene domains involved in each Moiré superstructure w.r.t. non-deformed graphene at the equilibrium distance on Pt(111).

On one side, the charge transfer occurring in each Gr/Pt(111) Moiré has been calculated by means of a Bader analysis^47^. Results of this analysis are summarized in Table 1. The charge transfers obtained are 0.0057, 0.0067, 0.0073, 0.0106 and 0.0114 e^−^ per C atom for the 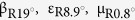, 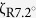, and 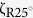 Moirés, respectively, always occurring from the Pt substrate towards the graphene, which manifests the *n*-doped character of the graphene layer in all the superstructures, as reported previously^44^. These values for the charge transfer for all the domains scale with the average weighted adsorption distances (see Table 1).

On the other hand, in order to estimate the Dirac-cone *left-shift* (and the local *n*-doping level) by effect of the formation of the different Moirés, we have designed a theoretical procedure consisting in obtaining the equilibrium geometries of flat graphene on Pt(111) (forcing the graphene layer to keep flat, but with the possibility of relaxing the G-Pt perpendicular distance) for each periodicity analyzed in this study. In this way, one can just integrate downwards the PDOS profile from the Fermi energy up to a point in the density of states profiles fulfilling the condition of integrating just up to the charge transfer obtained by the Bader analysis for all the cases. This theoretical strategy permits a direct comparison between the projected DOS on flat graphene on Pt for every domain with the projected DOS on graphene on Pt once the Moirés have been formed. By construction, this direct comparison yields the Dirac-cone shift by effect of just the formation of the different corrugated Moirés, eliminating in the shift values so obtained any possible contribution coming from the tendency of Gr and Pt chemical potentials to align.

**Figure 5:**
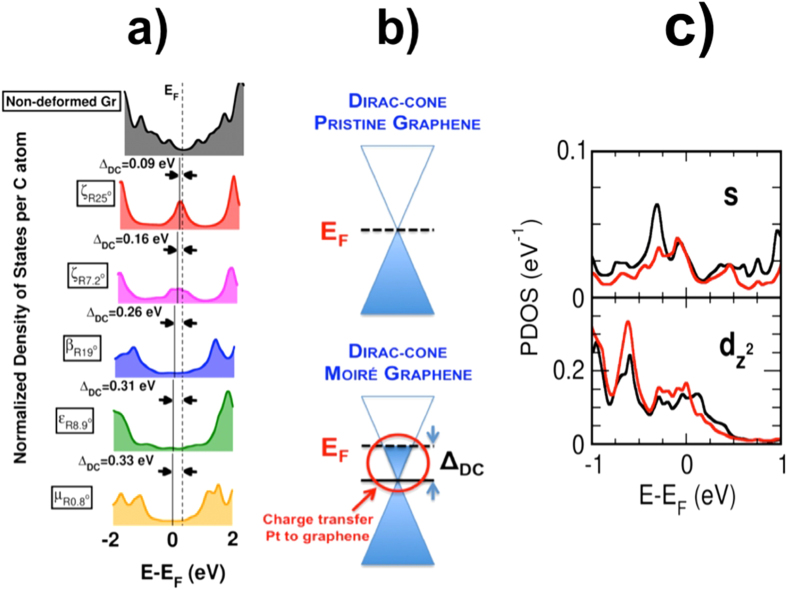
(**a**) Calculated DOS (in an energy window of [−2, 2] eV) projected on graphene (PDOS) involved in each Gr/Pt Moiré of Fig. 1, as well as for non-deformed graphene on Pt(111). For a better comparison, all the DOS profiles have been normalized to the number of C atoms in each unit cell. (**b**) Scheme of the Dirac-cone for pristine graphene and for graphene in each Gr/Pt, representing the *left-shift* of the Dirac-cone to accommodate the charge transferred from the Pt substrate. (**c**) Projected density of states (PDOS) on the *s* and 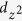 orbitals of a Pt atom forming a *pinning-point* in the 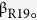 Gr/Pt system (red line) as compared to a surface Pt atom of the clean Pt(111) surface (black line).

Figure 5 shows the calculated DOS, in an energy window of [−2, 2] eV, projected on graphene involved in each Gr/Pt Moiré, as well as for their non-deformed graphene on Pt(111) forms as described above (see top panel of Fig. 5a for the unaltered graphene on Pt(111) rotated 19° as an example). For a better comparison, all the DOS profiles have been normalized to the number of C atoms. In Fig. 5a, one observes how the Dirac-cone *left-shifts* for all the different configurations (see Fig. 5b). This observed *left-shift*, labelled in Table 1 and Fig. 5 as Δ_*DC*_ (in eV), may be explained considering that the DOS moves towards lower energies to make available some of the empty states in the non-deformed graphene to accommodate the local excess of electronic charge coming from the Pt substrate in each Moiré with the respect the Fermi level of the interacting systems, in agreement with previous results^45^. For the cases of the 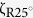 and 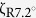 Moirés, which exhibit the heaviest Gr and Pt corrugations, respectively, an electronic state is induced around the Fermi energy, which permits accommodate their largest charge transfers from the Pt (see Table 1) showing small Dirac-cone shifts w.r.t. their non-deformed graphene forms, of 0.09 and 0.16 eV. Nevertheless, in the rest of cases, this induced electronic state does not appear, and the Dirac-cone *left-shift* is more pronounced, of 0.26, 0.31 and 0.33 eV for 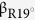, 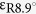 and 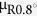, respectively, in order to accommodate the transferred charge from the substrate into their “new” available electronic states. It is important to notice that the Dirac-cone shift values scale with the charge transfer occurring from Pt towards Gr for these 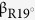, 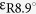 and 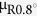 structures. Meanwhile, the domains exhibiting the highest charge transfers, 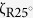 and 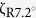, do not show such correlation. This behavior may be explained in terms of the essentially different structural “internal” morphology of each *pinning-point*. The *pinning-points* in the side of the Pt substrate for the 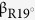, 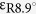 and 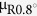 Moirés end with just a Pt atom at the cusp; whilst the *pinning-points* for the 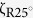 and 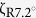 domains exhibit a three Pt atoms termination at the edge, the three almost at the same height forming a sort of small plateau. This particular configuration for the 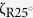 and 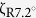 configurations favors the formation of a surface state, able to accommodate the charge transfer in the graphene, not present in the other three configurations (see Fig. 5). This fact manifests that the interaction between Pt and Gr is more complex than that *a priori* one could think, and demonstrates that many structural and electronic factors participate in such particular interaction.

The concept of pinning points for explaining epitaxial graphene overlayers on Pt(111) can be extended to other transition metal surfaces, although the interaction area can be different. On Ru(0001) a small relaxation of the outermost metal layer has been found, and the interaction area around *the pinning-points* extends to 3 near-neighbours^6^. Recently, the case of Gr/Rh(111) has been also deeply analysed by Martín-Recio *et al.*^48^, and although exact values are not provided by the authors, the contact/interaction area is quite significant. The reason for this simple model to work can be related to the Pt malleability when interacting with carbon. Formation of Pt atomic vacancies have been proposed to be induced at the interface between a carbonaceous layer and some transition metal surfaces^44^,^49^, and we can speculate that the softer surfaces to carbon could be prone to form well-localized *pinning-points*.

Moreover, it is important to have in mind that our DFT-based model imposes commensurability, which may be absent in the real system. Nevertheless, the low mismatch between substrate and graphene makes that the atoms at the *pinning-points* are still the closer to each other, and that they will be the point from where to inject charge until the separation between C and Pt atoms stop the coherence of the Moiré.

An additional analysis of the projected density of states carried out on the substrate Pt atoms forming the *pinning-points* in all the Moiré superstructures reveals the origin of the non-negligible charge transfer in all the configurations. The mechanism underlying these interface interactions can be understood in terms of the increase of the chemical overlapping between the Pt substrate and graphene, which is caused by a migration of electronic charge from the *s* orbitals in the Pt atoms at the *pinning-points* towards their own 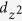 orbitals. Figure 5c shows this effect for the case of the 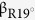 Gr/Pt system, where is possible to appreciate the s → 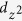 migration of electronic charge occurring at a Pt atom forming a *pinning-point* as compared with a Pt atom of a clean Pt(111) surface. This enhances the orbital perpendicular orientation in the Pt atoms, favoring the hybridization with the *p*_*z*_ orbitals from the Gr C atoms at the *pinning-points*. Those *p*_*z*_ orbitals arise from the emerging sp^3^ character of the C atoms at the *pinning*-*points* as a result of the pristine graphene sp^2^ hybridization rupture by the Gr buckling just at those *pinning-points*. It is important to mention that, very recently, hybridization between 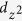 orbitals in the metal and *p*_*z*_ orbitals in the Gr has been proposed by Vita *et al.* as origin of band-gap formation in the Gr/Cu/Ir(111) interface^46^, which reinforces our interpretation about the localized *n-*doping mechanism in Gr/Pt(111) proposed here.

## Summary and Conclusions

Summarizing, we have shown that the structural and electronic properties of graphene superstructures on Pt(111) surfaces are dictated by the existence of *pinning-points*, which can be understood as the best coincident atoms between substrate and overlayer for a given rotational angle. At these *pinning-points* there is a significant atomic relaxation approaching the C and Pt atoms. As the distance is shorten, migrated electronic charge from the *s* towards the 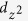
orbitals in the Pt atoms increases the orbital directionality, which favours the hybridization with the emerging *p*_*z*_ orbitals from the buckled Gr C atoms at the *pinning-points*. Thus, every one of the Moiré domain superstructures manifests a different *n*-doping and, therefore, the multidomain graphene/Pt(111) can be regarded as true graphene-laboratory for testing fundamental properties of doped graphene.

## Additional Information

**How to cite this article**: Martínez, J. I. *et al.* Role of the Pinning Points in epitaxial Graphene Moiré Superstructures on the Pt(111) Surface. *Sci. Rep.* **6**, 20354; doi: 10.1038/srep20354 (2016).

## Figures and Tables

**Table 1:** 

***Model***	***a***	***θ***^***rot***^	***θ***^***app***^	***d***	***C***^***Gr***^	***C***^***Pt***^	***E***^***ads***^	***PP***_***C-Pt***_	***ρ***_***t***_	***Δ***_***DC***_
***μ***_***R0.8°***_	21.0	0.8	6.6	3.19	0.13	0.02	0.25	3.15	0.0073	0.33
***ζ***_***R7.2°***_	15.4	7.2	9.0	3.14	0.38	0.12	0.28	2.83	0.0106	0.16
***ε***_***R8.9°***_	13.7	8.9	0.0	3.21	0.36	0.14	0.29	2.80	0.0067	0.31
***β***_***R19°***_	7.4	19.1	19.1	3.23	0.23	0.28	0.25	2.90	0.0057	0.26
***ζ***_***R25°***_	15.4	25.0	9.0	3.13	0.26	0.34	0.24	3.02	0.0114	0.09

Lattice parameter, *a* (in Å), rotation and apparent angles, *θ^rot^* and *θ^app^* (in °), weighted Gr—Pt perpendicular distance, *d* (in Å), Gr and Pt structural corrugations, *C^Gr^* and *C^Pt^* (in Å), adsorption energy per C atom, *E^ads^* (in eV/C), C—Pt distance at the *pinning-point*, *PP_C-Pt_* (Å) charge transfer per C atom from the Pt to the Gr, *ρ_t_* (in e^−^/C), and graphene Dirac-cone shift w.r.t. non-deformed graphene on Pt(111), *Δ_DC_* (in eV), for all Moiré superstructures shown in Fig. 1.
